# Behavioural physical activity interventions in participants with lower-limb osteoarthritis: a systematic review with meta-analysis

**DOI:** 10.1136/bmjopen-2015-007642

**Published:** 2015-08-10

**Authors:** Wilby Williamson, Stefan Kluzek, Nia Roberts, Justin Richards, Nigel Arden, Paul Leeson, Julia Newton, Charlie Foster

**Affiliations:** 1 Division of Cardiovascular Medicine, Cardiovascular Clinical Research Facility, University of Oxford, Oxford, UK; 2 Nuffield Department of Orthopaedics Rheumatology and Musculoskeletal Sciences, University of Oxford, Oxford, UK; 3 Information Specialist Department, Bodleian Health Care Libraries, University of Oxford, Oxford, UK; 4 School of Public Health and Charles Perkins Centre, University of Sydney, Sydney, Australia; 5 Nuffield Department of Population Health, British Heart Foundation Centre on Population Approaches for Non-Communicable Disease Prevention, University of Oxford, Oxford, UK

**Keywords:** RHEUMATOLOGY, SPORTS MEDICINE, REHABILITATION MEDICINE

## Abstract

**Objective:**

To assess effectiveness of osteoarthritis interventions to promote long-term physical activity behaviour change.

**Design:**

A systematic review and meta-analysis. Protocol registration PROSPERO CRD4201300444 5 (http://www.crd.york.ac.uk/prospero/).

**Study selection:**

Randomised controlled trials (RCTs) comparing physical activity interventions with placebo, no/or minimal intervention in community-dwelling adults with symptomatic knee or hip osteoarthritis. Primary outcomes were change in physical activity or cardiopulmonary fitness after a minimum follow-up of 6 months.

**Data extraction:**

Outcomes were measures of physical activity (self-reported and objectively measured) and cardiovascular fitness. Standard mean differences between postintervention values were used to describe the effect sizes.

**Results:**

27 984 titles were screened and 180 papers reviewed in full. Eleven RCTs satisfied inclusion criteria, total study population of 2741 participants, mean age 62.2. The commonest reasons for study exclusion were follow-up less than 6 months and no physical activity measures. The majority of included interventions implement an arthritis self-management programme targeting coping skills and self-efficacy. Seven studies used self-report measures, the pooled effect of these studies was small with significant heterogeneity between studies (SMD 0.22 with 95% CI −0.11 to 0.56, z=1.30 (p=0.19) I^2^ statistic of 85%). Subgroup analysis of 6–12 month outcome reduced heterogeneity and increased intervention effect compared to control (SMD 0.53, 95% CI 0.41 to 0.65, z=8.84 (p<0.00001) I^2^ of 66%).

**Conclusions:**

Arthritis self-management programmes achieve a small but significant improvement in physical activity in the short term. Effectiveness of intervention declines with extended follow-up beyond 12 months with no significant benefit compared to control. The small number of studies (11 RCTs) limited ability to define effective delivery methods. Investigation of behavioural lifestyle interventions for lower limb osteoarthritis populations would benefit from consensus on methodology and outcome reporting. This includes use of validated physical activity reporting tools and planning for long-term follow-up.

**Strengths and limitations of this study**

- To the best of our knowledge, this is the first systematic review of the longitudinal effectiveness of interventions to increase and maintain physical activity in lower limb osteoarthritis (OA) populations. A comprehensive search of several databases and sources was undertaken to identify eligible trials.
- We reduced potential bias in the conduct of this review by having authors independently screening titles and abstracts to identify a shortlist of full papers that was agreed for critical appraisal.
- Inclusion criteria for this study were rigorous, with emphasis on duration of follow-up and measurement of sustained behaviour change.
- The primary objective focused on assessing physical activity outcomes in defined OA populations. This improves homogeneity across the included studies but may create limitations for clinical translation.
- The meta-analysis should be interpreted with caution secondary to the identified heterogeneity and inherent risk of Simpson's paradox and associated ecological fallacy. This review did not evaluate cost-effectiveness of the interventions.

## Background

The lifetime risk of symptomatic lower limb osteoarthritis (OA) approaches 45% and generates a significant population health burden.^1–3^ OA is associated with increased prevalence of cardiovascular risk factors and excess mortality.^4^ ^5^ Morbidity in the obese OA population is equivalent to over a twofold increased risk of cardiovascular disease compared to non-obese OA free populations.^5–7^ The risk of OA is also associated with impaired glucose, hypertension and elevated cholesterol.^8^ Adipose tissue inflammation is a common link between OA and cardiovascular risk supporting a common aetiology between metabolic syndrome and symptomatic lower limb OA.^9–19^ One in two people with symptomatic OA are obese and the majority are inactive with less than 13% achieving recommended physical activity guidelines.^1^ ^20^ ^21^ Physical activity participation is independent of pain or radiological severity of OA.^22^ A criticism of current OA management is that it may be too reductionist, focusing on short-term musculoskeletal goals and neglects long-term behavioural outcomes.^23^ ^24^ Targeting modifiable behavioural risk factors such as physical activity may improve long-term morbidity and mortality.^6^ ^23–26^

## Rationale

Exercise intervention is an effective strategy for managing OA symptoms and immobility. The most recent systematic reviews identify a bias towards evaluating short-term pain, functional and well-being outcomes.^23^ ^27^ To date there has been no evaluation of the sustained effects of exercise intervention on physical activity behaviours or cardiovascular fitness exclusively in lower limb OA populations.^28^ ^29^

It is not clear if recommendations for best practice in lifestyle behaviour change are being incorporated into OA exercise intervention design and implementation.^30–32^ Recommendations includes: using validated measures of physical activity behaviour including questionnaires and activity monitors; targeting established guidelines for activity; using established behavioural theory as a framework for the interventions; measuring determinants of behaviour change; and, reporting outcomes beyond the termination of the behavioural intervention.

The objectives for this review are:

1. Evaluate effectiveness of OA behavioural interventions on sustained physical activity or cardiovascular fitness, over a minimum of 6 months, in lower limb OA populations.
2. To critically evaluate physical activity research methodology applied in randomised control trials of behavioural interventions for lower limb OA.
3. Summarise physical activity behavioural change strategies incorporated in OA exercise interventions for lower limb OA.

## Methods

### Protocol registration

PROSPERO (http://www.crd.york.ac.uk/PROSPERO/) No CRD42013004445.

### Search methods for identification of studies

An information specialist developed the search strategy based on an established design used in Cochrane reviews of interventions to promote physical activity.^33^ ^34^ The search terms spanned the breadth of exercise, lifestyle and physical activity descriptors and included trial and intervention specific terms. The updated search included osteoarthritis and musculoskeletal specific terms. The objective of the search was to be inclusive of populations with potential osteoarthritis burden in the general adult population exposed to exercise intervention. The search strategy is detailed in the supplementary online file. We searched the following databases: CENTRAL (Inception to June 2014), MEDLINE (1946 to June 2014), EMBASE (1974 to June 2014), CINAHL (1982 to June 2014), AMED (1985 to June 2014), PsycINFO (1967 to June 2014), SPORTdiscus (1980 to June 2014), OpenGrey (October 2012 and June 2014), SCISearch (1945 to June 2014), ACM Digital Library (October 2012 and June 2014) and IEEE Xplore Digital Library (October 2012 and June 2014). The Cochrane highly sensitive search was used to identify randomised controlled trials. No language restrictions were applied. The bibliographies of relevant review articles and selected articles were examined for additional potentially relevant trials. Literature searches were completed October 2012 and updated June 2014 with publications dates screened up to the 17 June 2014.

### Study inclusion criteria and selection

Two authors (WW and SK) independently manually screened the titles identified during the search to exclude those that were obviously outside the scope of the review. The authors were conservative at this stage and where disagreement occurred the citation was included for abstract review. Two authors (WW and SK) independently reviewed the abstracts of all citations that passed the initial title screen. The following inclusion criteria were applied to determine if the full paper needed further scrutiny.

Did the study:

1. Aim to examine the effectiveness of an exercise/physical activity/cardiovascular fitness promotion strategy?
2. Include a participant population where the majority had symptomatic, physician diagnosed and radiological confirmed diagnosis of OA?
3. Allocate participants in to the intervention or control group using a method of randomisation?
4. Have a control group that is exposed to placebo, no and/or minimal intervention?
5. Include adults of 16 years and older?
6. Recruit community dwelling adults?
7. Have a follow-up period of at least 6 months between start of the intervention and measuring the outcomes?
8. Analyse the results by intention-to-treat or, failing that, ensuring that there is less than a 20% loss to follow-up?

The authors were conservative and where disagreement occurred the citation was included for full text review. Two authors (WW and SK) reviewed the full text of all studies that passed the abstract screening using the inclusion criteria described above to identify the final set of eligible studies. When there was disagreement at this stage it was resolved after discussion with other authors (CF and JN). We linked publications and reports that utilised the same data to avoid replication in the analysis.

### Data collection and management

The data extraction form was independently piloted by two authors (CF and JR) and subsequently adjusted to ensure it captured the relevant data. Two authors (WW and SK) independently extracted the data from all the selected studies using the standard form. When there was disagreement a third author reviewed the study and a consensus was reached. We separately extracted data from multiple publications of the same study and then combined them to avoid replication. Any missing or ambiguous data was clarified with the study corresponding author.

### Assessment of risk of bias in included studies

The risk of bias was only assessed and reported for studies that met the inclusion criteria.^35^ The Cochrane Risk of Bias assessment instrument was expanded to include risk of bias assessment specific for physical activity interventions.^34^ Two authors (WW and SK) assessed the risk of bias. Where there was disagreement between review authors in the risk of bias assessment, a third author (CF or JN) was asked to independently appraise the study and discrepancies were resolved by consensus between all three authors.

We assessed the studies for the five general domains of bias: selection, performance, attrition, detection and reporting. Risk of bias scores were allocated for:

1. Allocation sequence generation;
2. Allocation concealment;
3. Incomplete outcome data;
4. Selective outcome reporting;
5. Comparable groups at baseline;
6. Contamination between groups,
7. Validated outcome measures,
8. Outcome measure applied appropriately;
9. Final analysis adjusted for baseline PA levels;
10. Outcome assessment that was independent and blinded;
11. Intention-to-treat analysis

When sufficient information was available, each domain was identified as ‘high’ or ‘low’ risk of bias. When there was a lack of information or uncertainty over the potential for bias, we described the domain as ‘unclear’.^34^ We judged the studies overall as having a ‘low’, ‘medium’, or ‘high’ risk of bias given consideration of the study design and size, and the potential impact of any identified weakness noted in the table for each study. The assessment of risk of bias and quality of included RCTs was then summarised using the GRADE approach.^36^

### Summary measures of treatment effect and unit of analysis

Studies were analysed using the mean and SD of outcomes expressed in the original papers. We expressed the effect size using the standard mean difference between the postintervention values of the randomised groups. We used the outcomes reported after the longest duration of follow-up. When studies investigated multiple interventions, intervention arms inclusive of exercise where combined, including interventions separating aerobic and resistance exercise. Means and SD were calculated for the combined intervention arms according to the overall numbers within each arm using established approaches.^36^

If domains of activity were reported separately within a single study, when possible mean effects were pooled to provide a summary effect for the intervention, otherwise, self-reported leisure time activity was used as the outcome measure. To allow comparison with reported intervention effects from previous reviews, including interventions in the general adult population, effect sizes were described according to Cohen's classification of effect size small (0.2 to <0.3), medium (0.3 to <0.8 and large (>0.8).^36^) Effect sizes for the individual studies were plotted with associated error bars using forest plots. Statistically significant results were identified as CIs excluding a null effect and an α value for z<0.05.

### Dealing with missing data

We excluded studies that had a high degree of incomplete data (defined as having more than 40% incomplete data) during the risk of bias assessment or when it appeared that the missing data were likely to be associated with the reported intervention effect. We contacted the authors of potentially included studies if missing data were unclear or data had not been fully reported. Missing data were captured in the data extraction form and reported in the risk of bias table. In the current review meta-analysis did not require imputation of missing mean values or SDs.

### Assessment of heterogeneity

Heterogeneity was quantified and evaluated to determine whether the observed variation in the study results was compatible with the variation expected by chance alone.^36^ Heterogeneity was assessed through examination of the forest plots and quantified using the I^2^ statistic according to the type of outcome utilised. I^2^ statistic was graded according to Cochrane interpretation (>75% considerable/large heterogeneity). The meta-analysis was repeated for each of the following outcome measures: cardiopulmonary function (Peak VO_2_), accelerometer and self-report outcomes.

### Assessment of reporting biases

Given the small number of studies and number of trials reporting different outcomes measures formal assessment of reporting bias, plotting on funnel plot, was not performed. This decision was made in accordance with Cochrane guidance for assessing reporting bias as plotting less than 10 studies may not distinguish between chance findings and real asymmetry.^37^

### Data synthesis

Meta-analysis was restricted to the seven studies with self-reported physical activity outcomes. Studies only reporting cardiovascular fitness and objective measures were limited in number and were excluded from meta-analysis. Analysis was completed using established methods.^36^ Analysis was performed using Excel Microsoft software incorporating MetaEasy statistical software and RevMan V.5.2 statistical software.^38^ ^39^ The DerSimonian and Laird random-effects model was the default to incorporate heterogeneity between studies, the inverse variance method used to calculate the overall effect and SE.^40^ Meta-regression analysis was completed using the Wilson (2010) SPSS macro using IBM SPSS Statistics for Windows, V.22.0. Meta-regression was completed using a random effects model.^41^

## Results

**Figure 1:**
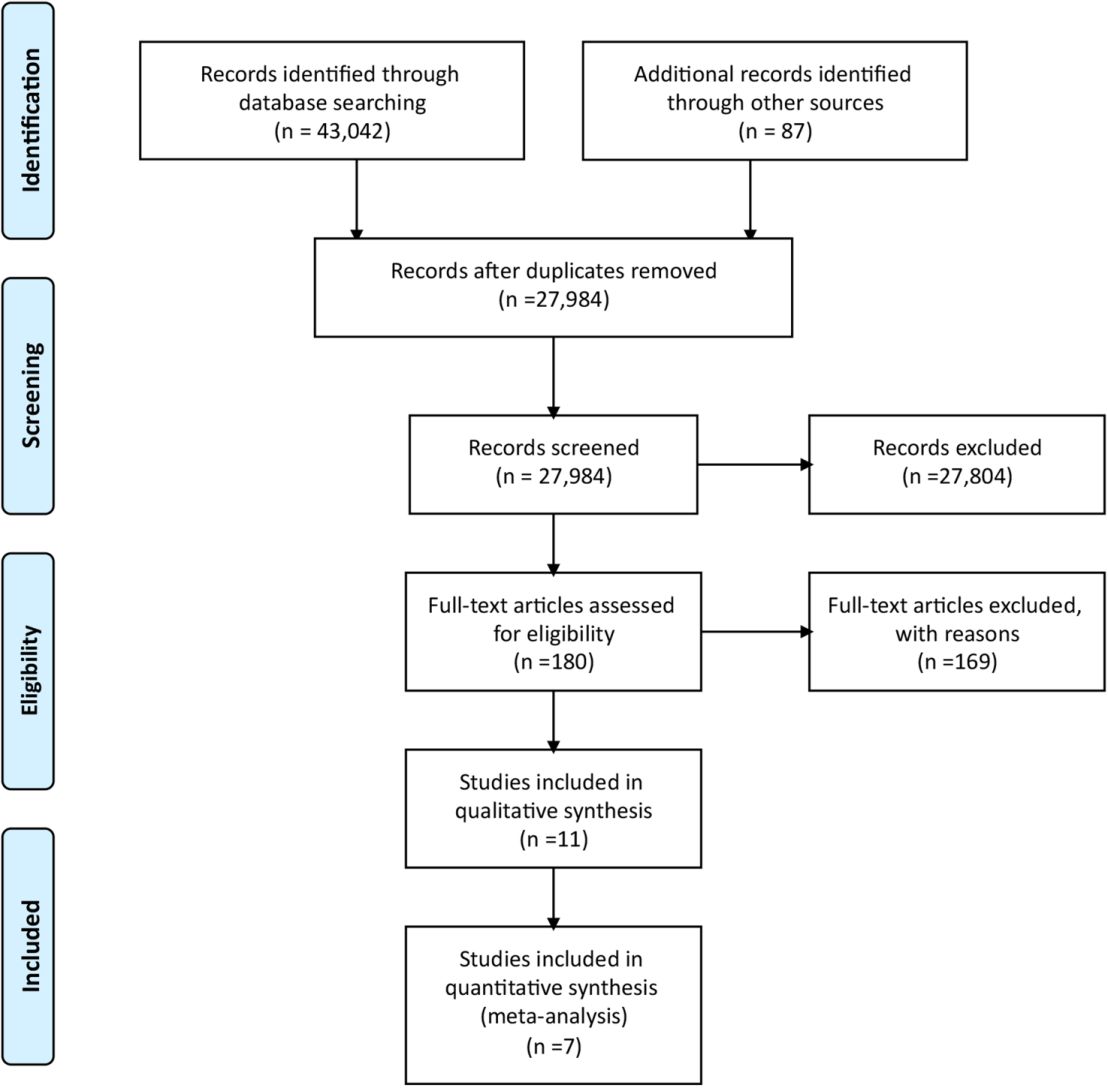
PRISMA flow diagram; randomised controlled trail study selection.

The literature search yielded 27 984 articles from across the physical activity and exercise literature. The majority of studies were excluded following review of titles and abstracts as not meeting the major inclusion criteria outlined in the study protocol. Of the 180 articles selected for critical reading (figure 1), 169 were excluded with explanation (see online supplementary file appendix 1). The majority of excluded randomised comparison trials report no or insufficient measures of physical activity (n=77).

Eleven studies (2741 participants, mean age 62.2) were included for review.^42–52^ Two studies were gender balanced while eight studies had over 70% female participation. One study which delivered an online intervention, relied on self-reported diagnosis of OA, physician diagnosis was confirmed in 68% of the population.^45^ The majority of interventions were completed in North America (n=8). The studies reporting ethnic demographic data (n=7) had study populations 70% White Caucasian. Full descriptions of the included studies and associated interventions and behavioural strategies are available in the online supplement (see online supplementary file appendix 2). Included trials were published between 1997 and 2013. The maximum length of follow-up was 29 months, the majority report between 6 and 12 months follow-up (8 trials). Six trials recruited participants with knee OA, four trials included hip and knee OA and one trial exclusively recruited participants with hip OA. Nine trials incorporate an arthritis self-management programme, targeting self-efficacy and coping skills. Five trials recorded a measure of self-efficacy as a potential determinant of behaviour change. Four (36%) trials discussed intervention design with context to Bandura's Social Cognitive Theory.^53^ ^54^ Two trials reported cardiopulmonary fitness, two reported accelerometer data, six trials used self-report measures of physical activity while one trial reported both accelerometer and self-report data. The majority of studies delivered the intervention within 6 months (n=8), utilising face-to-face interaction and supervised exercise, three interventions continued for between 9 and 18 months.

### Risk of bias and quality assessment

Risk of bias and additional quality markers were assessed across all included trials (see onlilne supplementary file appendix 3). The participant allocation methods were rated as low risk across all included trials. The majority of trials described randomisation at the individual level, one trial used cluster randomisation at the primary care practice level. Allocation concealment was adequate in 45% of the trials and not described in the remainder. Validated measures of physical activity or cardiopulmonary fitness were used in 9 of the 11 included studies. There was variability in the application of the outcome measures and methods applied with potential bias in accelerometer data collection. Bias primarily relate to the wear time and a deficiency in described strategies to improve participant compliance with wear time. All studies adjusted for baseline physical activity. In the majority of studies (7 of 11 studies) it was unclear whether outcome assessment was blinded. The greatest risk of bias related to incomplete data with increasing attrition across studies with duration of follow-up, 36% suffered attrition greater than 20% beyond 12 months, all of these studies included an intention-to-treat analysis. When available risk of selective reporting was assessed by comparing protocols or primary analysis plans with reported outcomes. In the majority of included studies selective reporting bias was low (90%). The overall assessment of the included RCTs using the GRADE approach suggests moderate quality data with majority of studies downgraded secondary to limitations in design.^36^

### Effects of interventions

#### Self-report measures of physical activity

**Figure 2:**
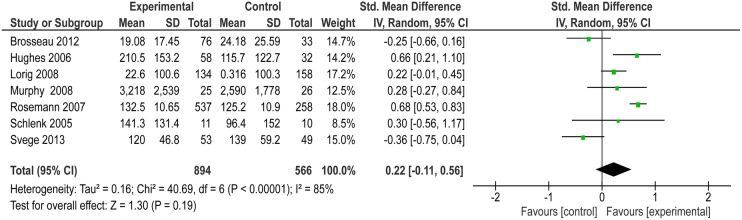
Forest plot for meta-analysis of self-reported physical activity outcomes following exercise intervention.

Seven studies reported physical activity outcomes using self-report measures, each study using a different measure. Five of the studies reported a positive effect, however only two studies reported a significant difference comparing intervention with control. The pooled effect for the seven interventions was not significant and there was considerable heterogeneity between the interventions (SMD 0.22 with 95% CI −0.11 to 0.56, z=1.3 (p=0.19) I^2^ statistic of 85%; figure 2).

All seven studies implemented an arthritis self-management strategy targeted to improve self-efficacy and four trials based interventions on Bandura's Social Cognitive Theory.^54^ Five of the studies combined arthritis self-management with supervised exercise. The intensity and duration of interventions varied across the studies (table 1).

**Table 1:** Intervention duration, participant-provider contact time, subsequent intervention compliance, attrition and self-report outcome over respective study duration

Study	Number of participants at baseline	Intervention duration	Estimated hours of contact	Follow-up duration months	Compliance	Attrition	Effect of intervention compared to control on physical activity outcome at follow-up				
							6m	9m	12m	18m	29m
Brosseau	222	12 months	178	18	77% 0–3 months 60% at 12 months	18% at 6 months 41% at 18 months	0	.	0	0	.
Hughes	215	8 weeks	36	12	70%	45.1% at 6 months 58% at 12 months	+	.	+	.	.
Lorig	292	6 weeks	78	12	Over 90%	23%	0	.	0	.	.
Murphy	54	6 months	14	6	Over 79%	0%	0	.	.	.	.
Rosemann	1021	6 month	Less than 7	9	Over 90%	22.10%	.	0	.	.	.
Schlenk	26	15 weeks	15	12	Over 90%	19%	+	.	+	.	.
Svege	109	12 weeks	36	29	75%	6%	0	.	0	0	0

(0) No significance difference in self-reported physical activity between intervention group and control, (+) significance difference in self-reported physical activity between intervention group and control, (.) no data.

Planned interaction between participants and the interventions ranged from less than 7–138 h. Compliance with the interventions calculated using mean participant attendance and presented as a percentage of all available intervention sessions/activity, was above 70% for all studies within the first 12 weeks of the intervention. Five of the studies completed 12 month follow-up, attrition was high for two for these studies above 40% at 12 months. Both studies applied an intention-to-treat analysis.

Post hoc meta-regression was completed to explore the influence of (1) Age of participants at baseline, (2) Estimated hours of contact with the intervention, (3) Duration of follow-up in months and (4) Rates of attrition, on effectiveness of intervention. In bivariate analysis estimated hours of contact time with the intervention and duration of follow-up had significant influence on intervention effectiveness. In multivariate regression only duration of follow-up remained significant (β coefficient for regression −0.04 (95% CI −0.08 to −0.004) p=0.03) with diminished effect of intervention at extended follow-up. To explore time effects a subgroup analysis was completed restricting meta-analysis to interventions reporting 6–12 months outcomes. This included five studies, total population of 1249 participants. The pooled standard mean difference between intervention and control was 0.53 (95% CI 0.41 to 0.65), z score=8.84 (p<0.00001) with I^2^ statistic of 66%. Included in this subgroup was the study by Lorig *et al*^45^ which delivered an online intervention. Recruitment for this trial relied on self-reported diagnosis of OA and only 68% of this cohort had a physician confirmed diagnosis. Excluding this study on the grounds of diagnosis improved the heterogeneity across the four remaining studies (n=957, SMD 0.64 (95% CI 0.51 to 0.78) z score=9.17 (p<0.00001), I^2^ statistic=0). The subgroup analysis supports a significant improvement in short-term physical activity up to 12 months following arthritis self-management programmes.

#### Objective measures of physical activity

Three of the 11 studies reported physical activity outcomes using accelerometers as objective measures of physical activity, all three studies implemented arthritis self-management interventions.^46^ ^49^ ^51^ Talbot *et al*^49^ investigated nurse prescribed individual walking plans supported by activity self-monitoring. The intervention was completed over 12 weeks with weekly contact during a structured education programme. The study reported a negative effect comparing intervention and control (SMD −0.64 95% CI −1.33 to 0.05). Farr *et al*^51^ implemented a high-intensity 9 month intervention with the option of three exercise sessions per week (SMD 0.29 95% CI −0.03 to 0.61). The intervention was inclusive of 12 weeks of structured education targeting coping skills and self-efficacy. Murphy^46^ reported data for both objective and subjective physical activity measures. The effect margin using objective measurement showed no real difference over using the questionnaire (SMD 0.07 95% CI −0.48 to 0.62 verses SMD 0.28 CI −0.27 to 0.84).

#### Cardiorespiratory fitness

Two studies examined the effect of their intervention on cardiorespiratory fitness.^43^ ^50^ Ettinger *et al*^43^ examined the effectiveness of supervised weekly resistance and aerobic exercise, the comparison group received health education. The exercise interventions covered 18 months in duration. The first 3 months were high contact with three sessions per week, followed by prescription of a personal exercise plan that was supported with home visits and telephone calls. The standardised mean difference for the intervention was 2.35 (95% CI 2.07 to 2.62), representing a positive large effect. Thorstensson^50^ evaluated a 6-week intervention consisting of 2×60 min supervised weekly sessions, plus daily home resistance exercise and 30 minutes walking per day. The comparison group received usual care consisting of three sessions with a physical therapist during the 6-month intervention. The mean effect of intervention over control was negative (SMD −0.19 95% CI −0.76 to 0.37). There are significant differences between these two studies, both in intensity of contact, duration of intervention, control group and follow-up time. Heterogeneity assessment reflects this with an I^2^ statistic of 98%. Neither Ettinger nor Thorstensson defined a theoretical or behavioural framework for their interventions.

## Discussion

### Physical activity measurement and research methodology in exercise and osteoarthritis interventions

This is the first systematic review to evaluate sustained physical activity change following behavioural OA intervention. The current review identifies significant deficiencies in use of validated physical activity measurement tools in OA interventions. A total of 180 papers were considered in full for this review. Of the 169 papers that did not meet inclusion criteria, short duration of follow-up (n=46) and lack of parameterisation of physical activity (n=77) are the most common explanation for study exclusion. This is despite availability of a number of validated self-report physical activity tools.^55^ ^56^ Explanation for under-reporting of physical activity behaviours in the excluded studies is unclear. A minority of the excluded trials published trial protocols which has prevented review of selective outcome reporting.

To date exercise and OA reviews have included trials with as little as 4–6 weeks follow-up. The average follow-up in the current review was 12 months. Epidemiological modelling studies suggest that outcome evaluation should continue beyond 5 years to accurately measure cost-effectiveness and health outcome.^57^ In practice extended follow-up may be limited by funding restrictions or compromised by study attrition.

This review highlights limited consensus in research methodology, especially in relation to measurement of physical activity. A weakness in many of the studies is the use of self-reported minutes of exercise, which may only capture activity in one physical activity domain (recreation and leisure).^55^ Using validated questionnaires as opposed to recall of active minutes per week or exercise diaries may facilitate more comprehensive data capture across activity domains.^56^

Objective measurement of physical activity with wrist worn accelerometers provide a feasible method to monitor daily activity and provide an opportunity for participants to self-monitor behaviour change.^58–61^ Incorporating wearable devices and self-monitoring may additionally improve assessment of intervention compliance and fidelity allowing evaluation of remote and low contact interventions. Studies in the current review use early examples of accelerometers and the technology and methodology since this timeframe has progressed significantly. Physical activity protocol design has evolved to support the wearing of wrist worn accelerometers to provide seven full days of activity measurement. However, this duration of wear time and associated number of data points need a suitable infrastructure to collate and analyse the data.^62^

This review suggests that consensus is required for the use of physical activity measures in behavioural lifestyle interventions. A major concern is that the OA research community are failing to measure physical activity as a baseline covariant. There is a strong argument that physical inactivity should be included in baseline demographic profiling of all chronic disease trials, similar in priority to recording hypertension, obesity, smoking and metabolic dysfunction.^31^

### Behavioural strategies and intervention delivery methods used to increase exercise and physical activity in osteoarthritis interventions

The majority of included studies implement a prescriptive approach to increase activity, following a defined timetable of supervised exercise. The benefit of this approach is a guaranteed exercise dose is received and supervision encourages compliance with the intervention. However, such delivery methods may not be economically feasible and may potentially fail to increase activity across domains (home environment, recreation and leisure, active transport and occupational activity) and may even decrease total activity. A number of interventions in the current review report a negative effect on maintained overall activity.

The majority of included studies are theoretically strong using defined behaviour change frameworks. These include arthritis self-management programmes based on Bandura's self-efficacy theory^54^ which aim to improve coping skills and self-determination to manage symptoms. As a result the studies adhere to established guidance for implementing behavioural interventions.^53^ ^61^ ^63^ ^64^ However, only a minority of trials measure the mediators of change in behaviour. As a result it is difficult to identify the active ingredients within the arthritis self-management programmes. Education, peer persuasion and self-monitoring were components of effective programmes but it is not clear how they shape the intervention process and outcomes. Measuring the mediators of change, which may be objective or subjective markers, may help to track the transition towards a defined behavioural outcome.^32^ ^65^

Although arthritis self-management was the common behaviour programme, there was considerable variability in the delivery and intensity of the interventions. It is not possible from the current evidence base to reliably distinguish which delivery strategy is most successful. The majority of interventions deliver concentrated programmes in less than 6 months with high contact between participant and provider. To improve translation into clinical practice, further investigation of effectiveness for remote versus face-to-face interventions and supervised versus self-directed interventions and the associated costs and benefits of each intervention, are required.

### Effectiveness of lower limb OA interventions to promote physical activity in comparison to interventions in the general population

The review identified a trend towards a small positive effect on increasing self-reported physical activity after 6 to 29 months follow-up. Effect of intervention is greatest in the first 12 months with a significant increase in physical activity compared to control in this time frame. The results are comparable to a Cochrane review of interventions in the general population using self-report physical measures which identified a positive moderate effect (SMD 0.28 95% CI 0.15 to 0.41, I^2^ 83.5%).^66^

One previous review discussed behavioural strategies and physical activity outcomes in a meta-analysis combining rheumatoid and OA interventions.^29^ The review reported an effect of 0.69 (95% CI 0.49 to 0.88) from control trials (n=23) but a limitation was the inclusion of studies with short follow-up (minimum of 4 weeks), introducing the risk of over estimating the true longitudinal effect of the exercise intervention. The meta-regression and subgroup analysis in the current review confirms intervention effectiveness declines with extended follow-up. The inclusion of the inflammatory arthritis population and distinctions in study inclusion criteria (single arm, before and after studies) prevent valid comparison with this review.

## Implications for research and practice

Arthritis self-management programmes may support successful strategies for promoting physical activity in OA populations. However, there remain significant barriers to translating the evidence base into clinical practice. Barriers to translation which need to be addressed include:

1. Establishing a consensus on research methods and outcome reporting.
2. Establishing the infrastructure and training required to promote essential components of the self-management programmes (education, coping skills, goal setting, self-monitoring, peer persuasion and individual feedback).
3. Defining optimal delivery and communication strategies (peer lead, health professional facilitation, face-to-face interventions, remote interventions).
4. Identifying the optimal duration and intensity of intervention programme (daily, weekly, monthly contact).
5. Investigating the longitudinal effectiveness of interventions on cardiovascular morbidity in OA populations.
6. Quantifying the cost-effectiveness of arthritis self-management interventions.

## Conclusion

OA is a musculoskeletal diagnosis associated with significant risk of cardiovascular disease and increased mortality. Promoting sustained increase in physical activity behaviour has the potential to achieve pain and symptom control and to prevent secondary complications. Despite a significant volume of research investigating exercise for OA management the evidence base is deficient in physical activity reporting and methodological rigour. Generating the evidence base to incorporate behavioural intervention into clinical management will require consensus in research design, outcome reporting and investment in multicentre trials with multidisciplinary teams.
